# One year follow-up on a randomized study investigating serratus anterior muscle and pectoral nerves type I block to reduced neuropathic pain descriptors after mastectomy

**DOI:** 10.1038/s41598-023-31589-6

**Published:** 2023-03-21

**Authors:** Eva M. Flores, Flavia V. Gouveia, Marcio Matsumoto, Tomás H. F. S. Bonacif, Mayra A. Kuroki, Geiza Fernanda Antunes, Ana Carolina P. Campos, Pedro P. Kimachi, Diego O. Campos, Claudia M. Simões, Marcelo M. C. Sampaio, Felipe E. M. Andrade, João Valverde, Alfredo C. S. D. Barros, Rosana L. Pagano, Raquel C. R. Martinez

**Affiliations:** 1 Division of Neuroscience, Hospital Sirio-Libanes, São Paulo, Brazil; 2 Anesthesiology Medical Center, Hospital Sirio-Libanes, São Paulo, Brazil; 3 Neuroscience and Mental Health, The Hospital for Sick Children, Toronto, ON Canada; 4 LIM/23, Institute of Psychiatry, University of Sao Paulo School of Medicine, Sao Paulo, Brazil; 5 Instituto de Ensino e Pesquisa, Hospital Sirio-Libanes, Rua Professor Daher Cutait, 69, São Paulo, SP 01308-060 Brazil

**Keywords:** Surgical oncology, Prognostic markers, Breast cancer, Neuroimmunology

## Abstract

Breast cancer is the second most common diagnosed type of cancer in women. Chronic neuropathic pain after mastectomy occurs frequently and is a serious health problem. In our previous single-center, prospective, randomized controlled clinical study, we demonstrated that the combination of serratus anterior plane block (SAM) and pectoral nerve block type I (PECS I) with general anesthesia reduced acute postoperative pain. The present report describes a prospective follow-up study of this published study to investigate the development of chronic neuropathic pain 12 months after mastectomy by comparing the use of general anesthesia alone and general anesthesia with SAM + PECS I. Additionally, the use of analgesic medication, quality of life, depressive symptoms, and possible correlations between plasma levels of interleukin (IL)-1 beta, IL-6, and IL-10 collected before and 24 h after surgery as predictors of pain and depression were evaluated. The results showed that the use of SAM + PECS I with general anesthesia reduced numbness, hypoesthesia to touch, the incidence of patients with chronic pain in other body regions and depressive symptoms, however, did not significantly reduce the incidence of chronic neuropathic pain after mastectomy. Additionally, there was no difference in the consumption of analgesic medication and quality of life. Furthermore, no correlation was observed between IL-1 beta, IL-6, and IL-10 levels and pain and depression. The combination of general anesthesia with SAM + PECS I reduced the occurrence of specific neuropathic pain descriptors and depressive symptoms. These results could promote the use of SAM + PECS I blocks for the prevention of specific neuropathic pain symptoms after mastectomy.

**Registration of clinical trial:** The Research Ethics Board of the Hospital Sirio-Libanes/Brazil approved the study (CAAE 48721715.0.0000.5461). This study is registered at Registro Brasileiro de Ensaios Clinicos (ReBEC), and ClinicalTrials.gov, Identifier: NCT02647385.

## Introduction

Breast cancer is the second most common cancer in women with a reported incidence of 19.3 million cases worldwide and 10 million cancer-related deaths^1^. A potentially debilitating problem that afflicts patients with breast cancer after surgery is the chronic pain after mastectomy, which affects 25–60% of the patients^2,3^, severely impacts the quality of life^4^, and is often comorbid with depression^5^.

Post-mastectomy pain syndrome or post-breast surgery pain syndrome has been described as a mixed syndrome that occurs 6 months after the procedure, characterized by persistent moderate pain with neuropathic characteristics located in the anterior thorax, axilla, and/or medial upper arm^6,7^. The chronic neuropathic pain has different characteristics depending on the surgical treatment or nerve damage^8,9^. The intensity of pain after surgery and consumption of analgesic medication have been reported to increase the risk of neuropathic pain^10^. The complexity and difficulty of its treatment could be because of neuroinflammation involving the release of inflammatory interleukins, such as interleukin (IL)-1 beta, IL-6, and IL-10^11^. “Plasmatic inflammatory markers could be used as prognostic predictors of pain in large interval of time^12^, it has been proposed that patients who developed neuropathic pain syndrome seem to have higher levels of IL-6 in plasma^13,14^. Also, IL-6 concentration is positively correlated with pain severity^13^, and is a risk factor for increased intensity of pain^15^. IL-1 Beta has been used as prognostic biomarker predictors for the severity of pain in breast cancer^16,17^. In the same line of thinking, inflammatory biomarkers have been considered effective potential biomarkers for depression^18–20^”.

Regional anesthesia has been suggested to play a protective role in the development of chronic neuropathic pain after mastectomy^21,22^. The serratus anterior plane (SAM) block and pectoral nerves block type I (PECS I) are safe and effective in breast surgery, resulting in excellent postoperative analgesia^23–25^. The combination of SAM and PECS I blocks promotes effective analgesia and reduction of intraoperative fentanyl and intravenous morphine usage during mastectomy with the axillary approach and reconstruction^26^.

Despite all the advances in regional anesthesia for breast cancer, the role of SAM and PECS I blocks in the development of chronic pain has not been evaluated. The present report describes a prospective follow-up study of a previously published study^26^ to investigate the presence of chronic neuropathic pain 12 months after mastectomy in patients who received general anesthesia only or general anesthesia with SAM + PECS I blocks. As secondary goals, the use of analgesic medication, quality of life, depressive symptoms, and possible correlations between plasma levels of interleukin (IL)-1 beta, IL-6, and IL-10 as predictors of pain and depression were evaluated. The rational for the choice of those outcome variables is that chronic pain in general could be exacerbated by neuropathic pain-generating mechanisms leading to both the physical and emotional suffering of patients that negatively impacts the quality of life.

## Methods

### Study design

A prospective follow-up study of a previously published randomized controlled clinical study was conducted^26^. The Ethics in Research Committee of the Hospital Sirio-Libanes/Brazil Platform approved the project (CAAE 48721715.0.0000.5461), it is registered at Registro Brasileiro de Ensaios Clinicos (ReBEC), ClinicalTrials.gov, Identifier: NCT02647385, and the date of registration is 06/01/2016. All methods were performed in accordance with relevant guidelines and regulations.

### Participants

The main inclusion criterion was patients enrolled in our previous study^26^. Female patients within the age range of 18–75 years, with American Society of Anesthesiology (ASA) physical status I or II, suitable for radical mastectomy with axillary node dissection and breast reconstruction, who provided written informed consent were included. Exclusion criteria for enrollment in the previous study were allergy to medications used in the study, history of mental disorders and chronic pain. The reason is that history of chronic pain is an independent risk factor for neuropathic chronic pain after mastectomy^27^. Additional exclusion criteria for this report was lost to follow-up.

### Interventions performed in the previous published study

In our previous study^26^, patients were randomly allocated to general anesthesia alone or general anesthesia with SAM + PECS I blocks.

#### Randomization

The block randomization method for the clinical study was designed to randomize subjects into 1 of the possible groups, i.e. group I: general anesthesia, group II: SAM + PECS-1 block. After generation of the randomization codes by the corresponding author, the allocation was registered in opaque sealed envelopes, and the patients were kept blind to this process. An anesthesiologist recruited participants from the ambulatory care unit of Hospital Sirio-Libanes.

#### General anesthesia

The patients received midazolam (7.5 mg) orally as a premedication 1 h before surgery. Anesthesia was induced using fentanyl (2–3 mcg kg^−1^), propofol (2–3 mg kg^−1^), and cisatracurium (0.1 mg kg^−1^) or rocuronium (0.6 mg kg^−1^). Anesthesia was maintained using sevoflurane (1.5–2.0%) and 50% oxygen delivered via a circle system. Additional fentanyl boluses were administered if necessary.

#### SAM + PECS I blocks

The SAM and PECS I blocks were performed by a team composed of three experienced anesthetists, as described previously^23,26,28^. Briefly, for SAM block the 12.5 MHz linear probe was positioned in the midaxillary line at the level of T5, and an in-plane needle was inserted into the fascia between the latissimus dorsi and the serratus anterior muscle for the injection of ropivacaine (20 mL of 0.375%). For the PECS I block, 10 mL of 0.375% ropivacaine was injected in the fascia between minor and major pectoral muscles. Needle position was confirmed using visualization of the separation of the layers with dispersion of the injected volume.

#### Postoperative analgesia

All patients received a standardized postoperative analgesic regimen consisting of metamizole (1 g every 6 h), ketoprofen (100 mg every 12 h), and patient-controlled analgesia (PCA) rescue with intravenous morphine at the end of the surgical procedure.

#### Scales

The patients were interviewed using the following scales:

1. Numeric Rating Scale (NRS) is a standard scoring system to assess immediate pain levels, ranging from scores 0 (no pain) to 10 (worst pain). Chronic pain can be defined as NRS score > 0 on a 0–10 scale at least 3 months after the surgical procedure^29^.
2. *Douleur Neuropathique* 4 (DN4) questionnaire accesses seven domains of neuropathic pain (i.e. burning, painful cold, electric shocks, tingling, pins and needles, numbness, and itching) and tree domains related to sensorial examination (hypoesthesia to touch, hypoesthesia to pinprick and pain caused by brushing)^30,31^. In this study, the surgical area, axilla, medial arm, breast, or chest wall were evaluated, and a score equal or greater than 4 points characterized neuropathic pain^32^.
3. Short-Form Health Survey (SF-36) is a health status questionnaire consisted of eight domains including functional capacity, physical aspects, general state of health, vitality, social aspects, pain, emotional aspects, and mental health ranging from zero (decreased quality of life) to 100 (increased quality of life).
4. Patient Health Questionnaire-9 (PHQ-9) is an instrument assessing nine depressive symptoms along with a functional health assessment. Scores range from 0 (none) to 27 (severe) to identify depressive states^33^.

#### ELISA

Blood samples were collected in EDTA-Vacutainer tubes before and 24 h after the surgical procedure, and the plasma levels of IL-1 beta, IL-6, and IL-10 were measured using commercial enzyme-linked immunosorbent assay (ELISA) kits (R&D Systems), as described previously^26^.

#### Sample size

The sample size for the clinical study was previously calculated and can be found in our previous publication^26^. In this study, patients that completed 1-year follow-up were included in the analysis. Power analysis for this new study was calculated based on using www.openepi.com, based on the results of Qian et al.^29^, who evaluated SAM block (lidocaine) versus control group (saline) and produced an effect size of 1.5 for NRS. Twenty patients per group were required to achieve significant results with an alpha of 0.05 and a beta of 90%. In this study, 22 patients were included in each treatment group.

### Current outcomes 12 months after surgery

#### Primary outcome

The primary outcome measure was evaluation of chronic neuropathic pain development 12 months after the mastectomy. All patients were evaluated by an anesthesiologist specialist in pain management. The diagnosis of neuropathic pain is based on clinical evaluation based on the history and physical examination to evaluate signs and symptoms. The DN-4 survey was used as a screening tool to better understand the chronic neuropathic pain after mastectomy and was performed as previous described^30^. The patients were evaluated using the Numeric Rating Scale (NRS) and the Douleur Neuropathique 4 (DN4) questionnaire Also, the incidence of pain in other body regions was performed.

#### Secondary outcomes

Demographic, clinical, surgical, and nonsurgical treatment data were collected. The current use of analgesic medication and the quality of life indicator of overall health status were evaluated using the Short-Form Health Survey (SF-36), and depressive symptoms were evaluated using the Patient Health Questionnaire-9 (PHQ-9). IL-1 beta, IL-6, and IL-10 levels before and 24 h after the surgical procedure were evaluated as predictors of pain and depression.

### Study design

Twelve months after the surgical procedure, the blinded patients included in the previous study^26^ were interviewed, by a blinded experimenter, with the NRS, DN4, SF-36, PHQ-9, and asked about the use of analgesic medication at that time set point. The plasma levels of IL-1 beta, IL-6, and IL-10 were correlated with the NRS, DN4, and PHQ-9 scales as predictors of pain and depression. All data were entered into the REDCap (Research Electronic Data Capture) database.

### Statistical methods

The Shapiro–Wilk test was used to investigate data distribution and showed that the data analyzed in this study presented with normal distribution. Demographic, quality of life (SF-36), and pain (NRS) data were analyzed using the Mann–Whitney test. Analgesic medication and neuropathic pain (DN4) were analyzed using Pearson’s chi-square test and corrected with Yates’ continuity correction where applicable. Depressive symptoms (PHQ-9) were analyzed using Cochran’s Q test. Correlations were analyzed using Pearson’s correlation coefficient. Statistical significance was set at p ≤ 0.05.

## Results

**Figure 1:**
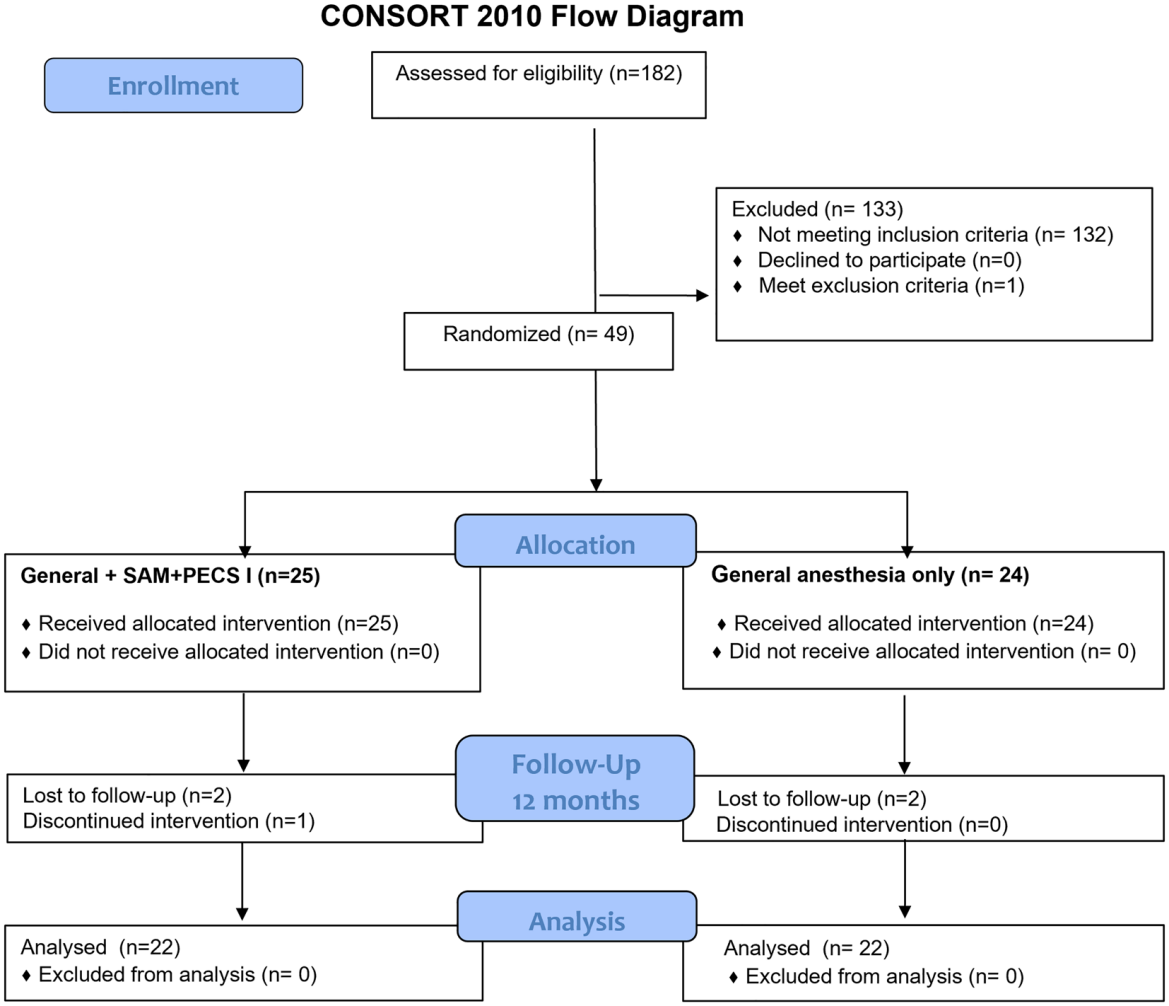
CONSORT flowchart of the surgeries performed during study development. General + SAM/PECS I: patients submitted to general anesthesia with serratus anterior muscle (SAM) block and pectoral nerves (PECS) block type I during mastectomy. General anesthesia only: patients submitted to general anesthesia only during mastectomy.

From 182 breast cancer surgeries performed between December 2015 and April 2016, 133 cases did not meet inclusion criteria because different types of surgery were performed including mastectomy only, lumpectomy or other breast-sparing surgery. A total of 49 patients were randomized and allocated to the Group II—general anesthesia with SAM + PECS I protocol (n = 25) or Group I general anesthesia only protocol (n = 24). For the follow-up in this study, which was performed 12 months after surgery, five patients were excluded. A total of 44 patients (22 in each group) were included in the analysis (Fig. 1).

### Baseline data

No complications related to nerve block procedure were reported.

There were no differences between the two groups in terms of age, body mass index, age at menopause, duration of mastectomy surgery, duration of the reconstruction procedure, and the number of lymph nodes removed, as presented in Table 1.

**Table 1:** Demographic, clinical, and surgical data.

Group	General anesthesia			General + SAM/PECS I			Mann–Whitney
	Median	25%	75%	Median	25%	75%	*p*
Age	52.0	48.0	64.0	57.0	46.0	67.0	0.488
Body mass index	26.0	23.9	28.3	27.8	25.9	29.4	0.260
Menopause age	48.0	43.0	50.0	51.0	40.0	52.0	0.308
Duration of the mastectomy (min)	90	77	120	87.5	60	100	0.319
Duration of the reconstruction (min)	180	103.8	240	180	117.5	241.3	0.802
Number of lymph node removal	13.0	9.0	20.0	22.5	4.0	27.0	0.165

Data showing in median and quartiles. General + SAMP/PECS I: patients submitted to the general anesthesia associated with serratus anterior muscle (SAM) block and pectoral nerves (PECS) block type I during mastectomy procedure (n = 22 patients). General anesthesia: patients submitted to general anesthesia only during mastectomy procedure (n = 22 patients).

There were no statistically significant differences between the two groups regarding the use of neoadjuvant therapy, neoadjuvant hormonal therapy, neoadjuvant trastuzumab, adjuvant therapy, adjuvant chemotherapy, adjuvant hormonal therapy, adjuvant trastuzumab, and adjuvant radiation therapy, as shown in Table 2.

**Table 2:** Data regarding the non-surgical treatment.

Group		General anesthesia		General + SAM/PECS I		X2
		*n*	%	*n*	%	*p*
Neoadjuvant therapy	No	12	54.5	11	50.0	0.763
	Yes	10	45.5	11	50.0	
Neoadjuvant hormonal therapy	No	20	90.9	22	100.0	0.469
	Yes	2	9.1	0	0.0	
Neoadjuvant trastuzumab	No	19	86.4	16	72.7	0.455
	Yes	3	13.6	6	27.3	
Adjuvant therapy	No	1	4.5	0	0.0	1.00
	Yes	21	95.5	22	100.0	
Adjuvant chemotherapy	No	11	50.0	16	72.7	0.122
	Yes	11	50.0	6	27.3	
Adjuvant hormonal therapy	No	4	18.2	3	13.6	1.00
	Yes	18	81.8	19	86.4	
Adjuvant trastuzumab	No	21	95.5	17	77.3	0.187
	Yes	1	4.5	5	22.7	
Adjuvant radiation therapy	No	6	27.3	6	27.3	1.000
	Yes	16	72.7	16	72.7	

General + SAMP/PECS I: patients submitted to the general anesthesia associated with serratus anterior muscle (SAM) block and pectoral nerves (PECS) block type I during mastectomy procedure (n = 22 patients). General anesthesia: patients submitted to general anesthesia only during mastectomy procedure (n = 22 patients).

### Primary outcome: pain

**Figure 2:**
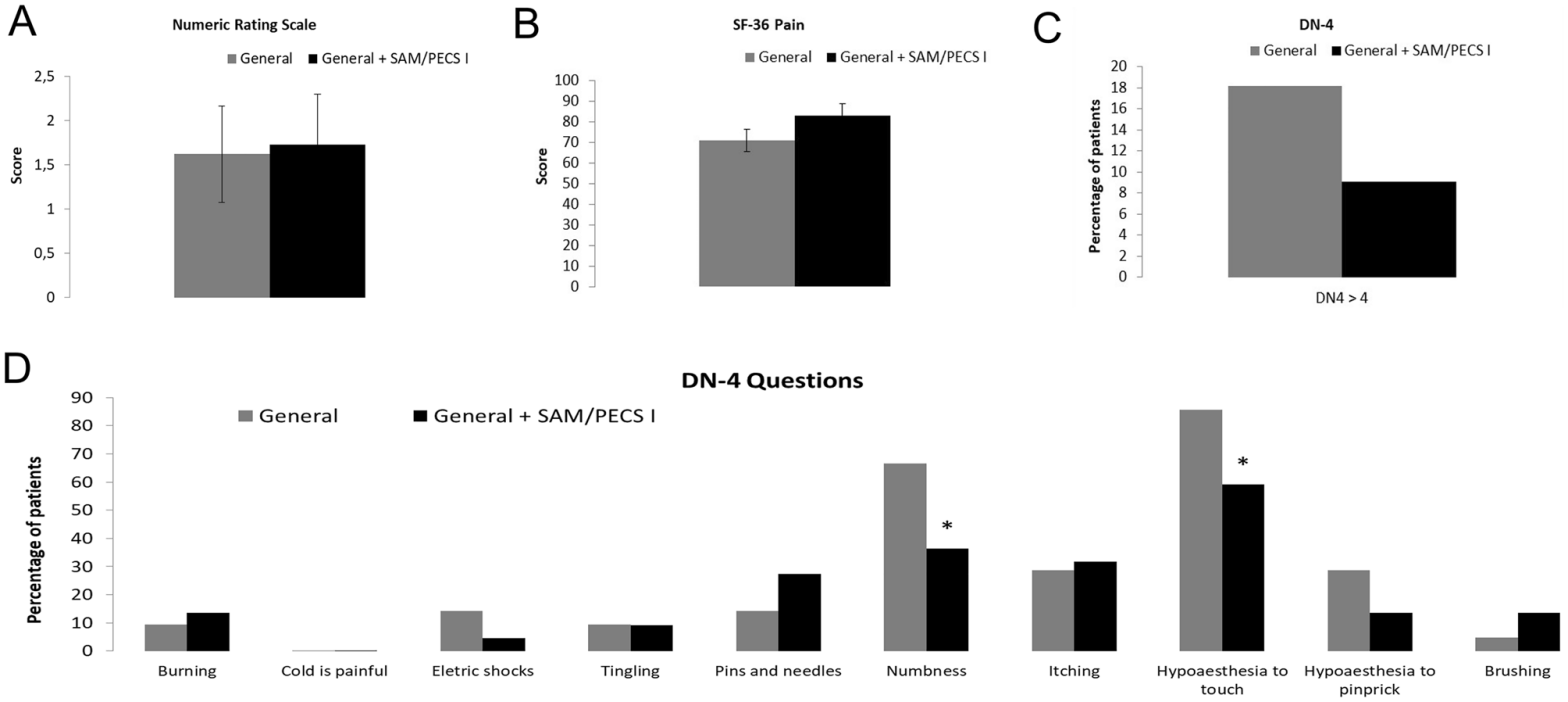
Pain scores and medication consumption in both experimental groups: general anesthesia (n = 22) and general anesthesia with SAM + PECS I blocks (n = 22). (**A**) Pain levels measured using the Numeric Rating Scale (NRS) 12 months after surgery. (**B**) Short-form Health Survey (SF-36) scores regarding the pain domain. (**C**) Percentages of patients reporting values above 4 on the Douleur Neuropathique 4 (DN4) scale. (**D**) Percentage of affirmative answers in the items of the DN4 questionnaire.

No significant difference (p = 0.54) was observed in the NRS pain scores reported by the two patient groups 12 months after mastectomy (Fig. 2A). Figure 2B presents the data regarding the pain subscale of the SF-36 scale 12 months after surgery, no significant difference (p = 0.138) was observed between groups.

Likewise, no significant difference (p = 0.379) was observed in the percentage of patients reporting DN4 punctuation > 4, as illustrated in Fig. 2C.

Figure 2D presents the percentage of affirmative answers for each of the 10 items in the DN4 questionnaire. No significant differences were observed in the domains burning (p = 0.674), cold in pain (p = 1.00), electric shocks (p = 0.272), tingling (p = 0.961), pins and needles (p = 0.295), itching (p = 0.295), hypoesthesia to pinprick (p = 0.229) and brushing (p = 0.317) between the groups. There was a reduction in numbness and hypoesthesia to touch in the Group II in comparison with the Group I (p = 0.047 and p = 0.049 respectively, Fig. 2D). Table 3 presents the comparison of the DN4 tool versus clinical evaluation for the probable chronic pain after mastectomy showing no difference between the evaluations.

**Table 3:** Comparison of the DN4 tool versus clinical evaluation for the probable chronic neuropathic pain after mastectomy.

	Probable chronic neuropathic pain after mastectomy		Statistics
	DN4 test	Clinical evaluation	p value
General	4/22	6/22	0.16
General + SAM/PECS I	2/22	3/22	0.32

Data regarding the number of patients that presented probable chronic neuropathic pain after mastectomy. General + SAMP/PECS I: patients submitted to the general anesthesia associated with serratus anterior muscle (SAM) block and pectoral nerves (PECS) block type I during mastectomy procedure (n = 22 patients). General anesthesia: patients submitted to general anesthesia only during mastectomy procedure (n = 22 patients).

### Primary outcome: incidence of chronic pain in other body regions

**Figure 3:**
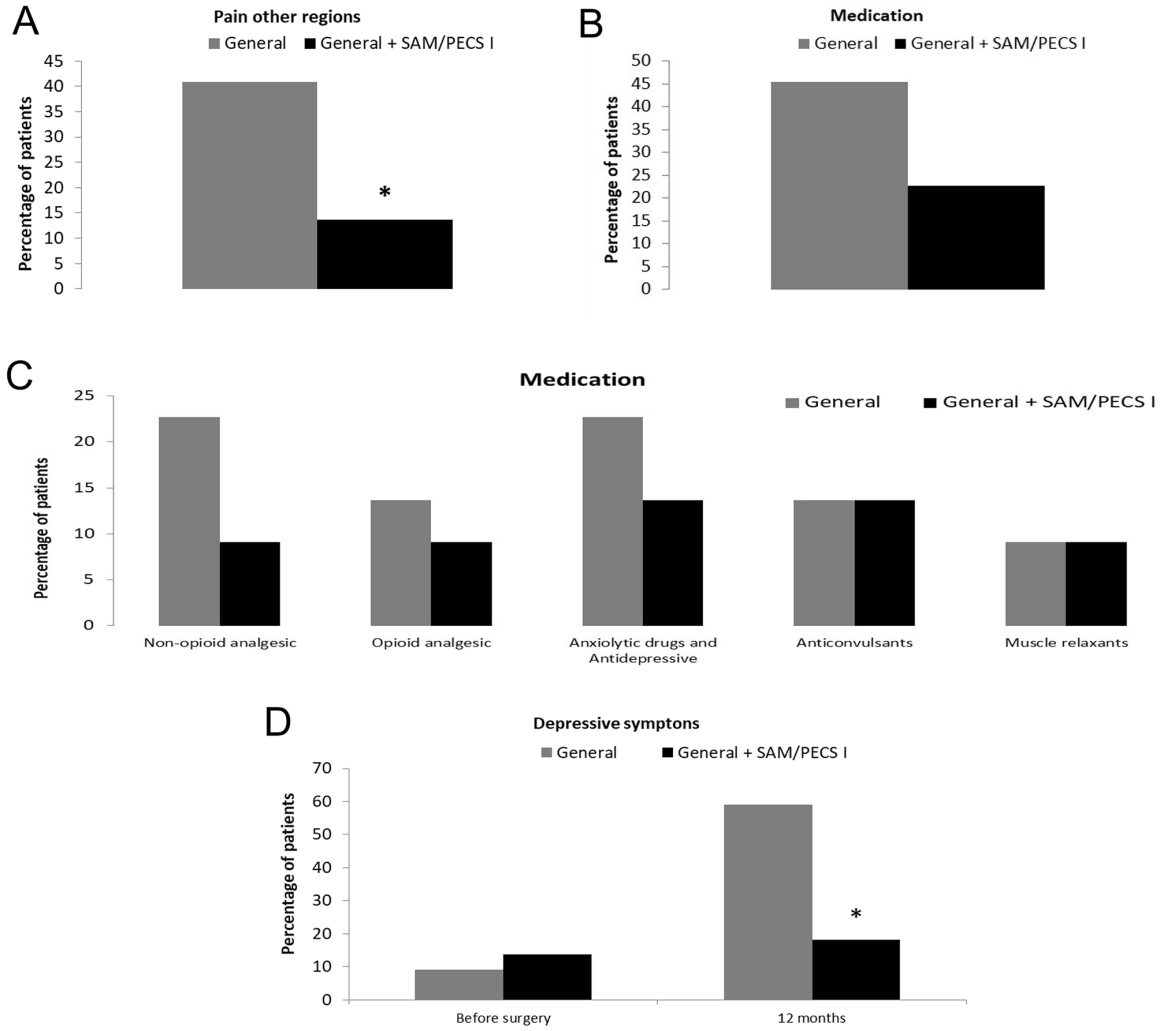
(**A**) Incidence of chronic pain in other regions. (**B**) Percentage of patients reporting the use of analgesic medication. (**C**) Percentage of patients reporting the use of nonopioid analgesics, opioid analgesics, anxiolytic drugs, antidepressants, anticonvulsants, and muscle relaxants. (**D**) Percentage of patients reporting depressive symptoms according to the Patient Health Questionnaire-9 (PHQ-9). The data are presented as the mean ± standard deviation or in terms of percentage of patients. *p < 0.05 compared with the general anesthesia-only group.

Group II showed a reduction in the percentage of patients showing pain in other body regions in comparison with Group I (p = 0.014), as shown in Fig. 3A. Also, Table 4 presents data regarding the clinical evaluation specifying the body region in which the chronic pain occurred.

**Table 4:** Data specifying the incidence of chronic pain in other body regions.

	General anesthesia		General + SAM/PECS I	
	*n*	*%*	*n*	*%*
Ankle	1	4.55	0	0
Legs	3	13.64	0	0
Hands	1	4.55	1	4.55
Low back	1	4.55	2	9.10
Hip	1	4.55	0	0
Forearm	1	4.55	0	0
Knee	1	4.55	0	0

General + SAMP/PECS I: patients submitted to the general anesthesia associated with serratus anterior muscle (SAM) block and pectoral nerves (PECS) block type I during mastectomy procedure (n = 22 patients). General anesthesia: patients submitted to general anesthesia only during mastectomy procedure (n = 22 patients).

### Secondary outcome: analgesic medication

No significant difference in the use of analgesic medication 12 months after the surgical procedure was noted between groups (p = 0.112), as shown in Fig. 3B. Figure 3C illustrates the data regarding the use of analgesic medications, categorized into five types according to their drug class. Among nonopioid analgesics, metamizole and paracetamol were included. No significant difference between groups were observed regarding the use of nonopioid analgesics (p = 0.4121), opioid analgesics (p = 0.99), anxiolytic drugs and antidepressants (p = 0.6981), anticonvulsants (p = 0.99) and muscle relaxants (p = 0.99).

### Secondary outcome: depressive symptoms

Data referring to the PHQ-9 scale are shown in Fig. 3D. There was no difference between groups before the surgery (p = 0.65). A lower percentage of patients allocated in Group II had depressive symptoms 1 year after surgery, compared with those who received general anesthesia only (p = 0.012).

### Secondary outcome: SF-36 domains

Table 5 presents the data from the SF-36 questionnaire 12 months after the surgical procedure. No significant differences between the two groups were observed in all aspects related to quality of life.

**Table 5:** Data obtained in the SF-36 domains including functional capacity, physical aspects, general state of health, vitality, social aspects, emotional aspects, and mental health.

Group	General anesthesia			General + SAM/PECS I			Mann–Whitney
	Mediana	25%	75%	Mediana	25%	75%	p
Functional capacity	82.5	60.0	90.0	80.0	55.0	85.0	0.637
Physical aspects	100.0	0.0	100.0	0.0	0.0	100.0	0.295
General state of health	55.0	50.0	70.0	60.0	50.0	65.0	0.878
Vitality	57.5	40.0	75.0	60.0	45.0	70.0	0.944
Social aspects	100.0	100.0	100.0	100.0	75.0	100.0	0.254
Emotional aspects	100.0	0.0	100.0	100.0	0.0	100.0	0.824
Mental health	72.0	60.0	80.0	70.0	64.0	84.0	0.714

Data showing in median and quartiles. General + SAMP/PECS I: patients submitted to the general anesthesia associated with serratus anterior muscle (SAM) block and pectoral nerves (PECS) block type I during mastectomy procedure (n = 22 patients). General anesthesia: patients submitted to general anesthesia only during mastectomy procedure (n = 22 patients).

### Secondary outcome: interleukins as the predictor of pain and depression

Table 6 presents the data correlating the plasma levels of IL-1 beta, IL-6, and IL-10 before and after the surgical procedure versus the pain score obtained in the NRS and DN4 questionnaires, and depression score obtained in the PHQ-9 1 year after surgery. No statistically significant correlations were observed between these parameters.

**Table 6:** Multiple regression data correlating the levels of interleukin before and after the surgical procedure with the pain score obtained in the Numeric Rate Scale (NRS), Douleur Neuropathique 4 (DN4) questionnaire, and with the depression score obtained in the Patient Health Questionnaire-9 (PHQ-9) 12 months after surgery.

Interleukin	Numeric rate scale (NRS)	Douleur neuropathique 4 (DN4)	Patient health questionnaire-9 PHQ-9
IL-1β before mastectomy	R^2^ = 0.0215, p = 0.34	R^2^ = 0.0041, p = 0.68	R^2^ = 0.0136, p = 0.55
IL-1β after mastectomy	R^2^ = 0.0263, p = 0.29	R^2^ = 0.0051, p = 0.64	R^2^ = 0.0171, p = 0.51
IL-6 before mastectomy	R^2^ = 0.0018, p = 0.78	R^2^ = 0.0138, p = 0.44	R^2^ = 0.0295, p = 0.38
IL-6 after mastectomy	R^2^ = 0.0021, p = 0.76	R^2^ = 0.0070, p = 0.58	R^2^ = 0.0281, p = 0.39
IL-10 before mastectomy	R^2^ = 0.0019, p = 0.77	R^2^ = 0.0139, p = 0.44	R^2^ = 0.0319, p = 0.36
IL-10 after mastectomy	R^2^ = 0.0001, p = 0.98	R^2^ = 0.0257, p = 0.29	R^2^ = 0.0282, p = 0.39

Data showing in R^2^ value and the corresponding p value.

## Discussion

To our knowledge, this is the first follow-up study evaluating chronic neuropathic pain after mastectomy in patients who were administered general anesthesia with SAM and PECS I blocks compared with those who received general anesthesia during the surgical procedure. The results showed that 12 months after mastectomy with axillary approach and reconstruction, the patients who received general anesthesia with SAM and PECS 1 blocks did not have a decreased incidence of chronic neuropathic pain; however, they presented a reduction in the descriptors numbness and hypoesthesia; and in the incidence of patients that present chronic pain in other body regions. Importantly, there was a reduction in depressive symptoms in the general anesthesia with SAM and PECS I blocks group. We had chosen not including different types of surgical procedures in our study to have a homogenous sampling and being able to further understand and describe^34^ the role of SAM + PECS1 focus on this subgroup of interest.

Demographic data showed no difference between the groups regarding age, body mass index, menopausal age, and duration of the surgical procedure. Our data agree with the median age at diagnosis of breast cancer in middle-aged and older women^35^. It is suggested that body mass index may be an important predictor of post-mastectomy chronic pain development^36^. The age of onset of menopause is important for guiding treatment strategies^37^. However, in the present study, these parameters did not influence the development of chronic pain. Regarding axillary lymph node dissection, which has been considered a major risk factor for chronic neuropathic pain after mastectomy^36^, our data showed no statistically significant difference between groups. Chemotherapy and radiation may lead to the development of chronic persistent post-surgical pain^38^. Also, the lymphatic system dysfunction could affect lymph drainage^39^ and contribute to chronic pain in general, functional impairment, and depression^40,41^. It is important to point that our data did not showed difference between groups in those parameters.

Additionally, the duration of surgery could increase the severity of acute pain and consequently, has been associated with persistent pain^42^. Therefore, it is important to emphasize that there were no significant differences between the two groups in terms of these parameters that could influence the development of chronic neuropathic pain after mastectomy.

Chronic neuropathic pain after mastectomy is caused by lesions or diseases of the somatosensory nervous system, which may lead to increased pain sensitivity, spontaneous pain, and loss of function^43^. Also, this type of chronic pain has no purposes of adaptation, devoid of biological value, not respond to typical treatment and last more than six months, causing suffering, distress and contributing to deteriorate the quality of life of the patients^44^. Several screening tools have been developed to identify possible symptoms of neuropathic pain owing to their varied manifestations^45^. The DN4 questionnaire is a valuable tool for investigating neuropathic pain profile and assessing its possible mechanisms^30,46^. DN-4 questionnaire has high sensitivity and specificity in distinguish chronic neuropathic pain from chronic nonneuropathic pain^47,48^ and is a reliable tool for evaluating chronic neuropathic pain and the effect of regional anesthesia as protecting strategies after breast cancer surgery^49^. Our study performed a detailed analysis of DN4 items to better understand the chronic neuropathic pain after mastectomy of these patients. Our data are in accordance with the literature, showing that hypoesthesia to touch is a sensory dysfunction, followed by numbness^30,50^, also there was a reduction in the incidence of patients reporting chronic pain in other regions. General anesthesia with SAM and PECS I blocks reduced the occurrence of hypoesthesia to touch and numbness compared with general anesthesia alone. A possible explanation could be that the patients who received SAM and PECS I blocks experienced a reduction in postoperative pain^26^. Chronic pain in general is associated with inadequate postoperative pain control^8^. In addition, the analgesic pattern offered by the SAM + PECS 1 blocks provide excellent analgesia to the breast and axillary regions^51–54^.

The use of the PECS block has been shown to induce a lower incidence of chronic postsurgical pain in general 3 months after breast surgery^55^. The use of SAM block was also shown to reduce its prevalence 6 months after mastectomy^29^, which is consistent with our findings. In the pathophysiology of chronic pain after mastectomy, the neuropathic categories are attributed to nerve damage or traction during surgery, particularly targeting the intercostobrachial, medial pectoral, lateral pectoral, thoracodorsal, and long thoracic nerves^6,56,57^. Thus, it has been postulated that nerve injury, neuroma, phantom breast pain, anatomic changes, axillary web syndrome, and lymphedema could be responsible for the neuropathic pain observed after breast surgery, and local anesthetic blockade of this region could be an alternative to prevent this type of pain^56^.

It is important to highlight that most of the studies that evaluated the development of chronic pain in general were performed with paravertebral blocks and demonstrated controversial results, showing pain reduction^58^ or no effect^59^. The analgesic pattern of the paravertebral block reaches the sympathetic and intercostal nerves, while the combination of SAM + PECS I blocks can reach the thoracodorsal and long thoracic nerves, which are thought to be involved in the pathophysiology of chronic post-mastectomy neuropathic pain^52^. The analgesic pattern of SAM and PECS I blocks could explain the reduction in neuropathic symptoms, such as numbness and hypoesthesia.

Our data showed no difference between the two groups regarding the SF-36 questionnaire domains. This tool has been routinely used in patients with breast cancer^60^ to evaluate quality of life and was used in the present study to identify possible confounding factors in the assessment of chronic pain.

The diagnosis and identification of depression can be challenging in view of the wide variety of symptoms presented by patients. Pain and depression are common symptoms exhibited after mastectomy^42,61^. Psychological determinants, such as depression, is associated with more pain complaints and greater intensity of pain^62^. The PHQ-9 scale has been reported as a useful tool in assessing depressive symptoms^33^. Our data showed that patients who received general anesthesia with SAM and PECS I blocks had a lower incidence of depressive symptoms. To our knowledge, there is no association between the use of regional anesthesia and depression. A possible explanation could be that pain and depression share the same neuroanatomical substrate including biological pathways and neurotransmitters and the presence of pain negatively affect the recognition of depression^62^. In this sense, the use of regional anesthesia could decrease the neuronal reorganization contributing to simultaneously reduce pain and depression^63^. Also, it could be proposed that the use of SAM and PECS I blocks contributed to decreasing postoperative pain^26^ and consequently reducing the negative impact of the surgical procedure on the psychological aspects^64^.

Because of the intrinsic clinical relationship between chronic pain in general and depression, this association has been called depression-pain syndrome or depression-pain dyad and has a direct impact on the quality of life and individual health care^65^. The mechanisms responsible for this depression-pain syndrome are not fully understood, but it is thought to involve neuronal reorganization in response to neuroinflammation in brain areas responsible for emotional processing and pain perception^63,66,67^.

Considering the consumption of analgesic medication after mastectomy, a previous study showed that approximately 40% of patients take analgesic medication for chronic pain in general^68^. These data are in accordance with our results regarding the group subjected to general anesthesia only, while the patients who received regional and general anesthesia had a lower percentage of analgesic medication consumption. However, there was no statistically significant difference between the two groups, probably because the sample size was calculated for acute pain^26^. Specifically, our patients had been using a wide variety of medications, including nonopioid analgesics, opioid analgesics, anxiolytics, antidepressants, anticonvulsants, and muscle relaxants. These data are in accordance with a systematic review focusing on analgesic medications for breast surgery, suggesting that anticonvulsants may decrease the incidence of chronic pain; however, the beneficial role of combinations of analgesic medications is not completely understood^69^.

Treatment therapies are important variables that contribute independently to the prediction of chronic pain in general^70^. Chemotherapy, hormone therapy, and radiotherapy have been proposed to be significantly associated with higher scores for the development of chronic pain^36^. Our results showed no differences in these parameters, supporting that the reduction in neuropathic descriptors in the regional anesthesia group was independent of the treatment therapies.

We performed correlation tests using the plasma levels of cytokines and the chronic pain and depression. The rationale for this correlation is that a sustained increase in cytokine levels could contribute to central sensitization that is responsible for the maintenance of chronic pain^71^. In addition, neuroinflammation could be responsible for the depletion of brain serotonin, dysregulation of the hypothalamus–pituitary–adrenal axis, and hippocampal neurogenesis alteration, which contribute to depressive symptoms. It has been shown that IL-6 has a pivotal role in the development of pathological pain^72^, IL-1 drives chronic pain^73^ and IL-10 could suppress proinflammatory cytokines that modulates chronic pain^74^. Also, considering depression, IL-6 and IL-10 could be a potential biomarker of prognosis and severity^75–77^, while IL-1 levels could predict probability of response to antidepressants^78^. However, in this study, no correlation was observed between the levels of IL-1 beta, IL-6, and IL-10 before and 24 h after surgery, and the NRS, DN4, and PHQ-9 scores obtained 12 months after surgery. A possible explanation for the lack of differences could be attributed to the fact that we evaluated plasma cytokines, which serve as biomarkers, but do not directly reflect the neuroinflammation process, which could be better visualized using the cerebrospinal fluid^79^.

Our current study had some limitations. First, we evaluated chronic neuropathic pain at a single time point. Second, sample size calculation was performed for acute pain and is relatively small but is important to highlight the homogeneity of our patient sample. Also, postoperative pain therapies were not standardized and could be a potential source of bias in the results. Future research could address these limitations.

In conclusion, our results suggest that the SAM and PECS I blocks associated with general anesthesia reduce the occurrence of two neuropathic pain descriptors, including numbness and hypoesthesia to touch and decrease the incidence of depressive symptoms after mastectomy.

## Data Availability

Data are available from the corresponding author upon reasonable request from the REDCap database.
